# Effects of Thyme (*Thymus vulgaris*) Essential Oil on Bacterial Growth and Expression of Some Virulence Genes in *Salmonella enterica* Serovar Enteritidis

**DOI:** 10.1002/vms3.70088

**Published:** 2024-10-30

**Authors:** Mohammad Hassanzadeh, Sara Mirzaie, Faezeh Rahimi Pirmahalle, Ramak Yahyaraeyat, Jamshid Razmyar

**Affiliations:** ^1^ Department of Avian Diseases Faculty of Veterinary Medicine University of Tehran Tehran Iran; ^2^ Department of Animal Poultry and Aquatics Institute of Agriculture Iranian Research Organization for Science and Technology (IROST) Tehran Iran; ^3^ Department of Pathobiology Faculty of Veterinary Medicine University of Tehran Tehran Iran

**Keywords:** essential oils, Salmonella enteritidis, Thymus vulgaris, virulence genes

## Abstract

**Background:**

The investigation on natural antimicrobial compounds against zoonotic pathogens has gained more attention due to the public health concerns regarding the emergence of antimicrobial resistance.

**Objectives:**

The current study aimed to assess the effects of thyme essential oil at sub‐minimal inhibitory concentrations (sub‐MICs) on bacterial growth and expression of some virulence genes in *Salmonella enteritidis*.

**Methods:**

The bacterial growth rate and the expression of four virulence genes in *S*. *enteritidis* during 18–72 h of exposure to the essential oil at 25%–75% MIC were evaluated via colony counting and real‐time polymerase chain reaction (PCR), respectively.

**Results:**

Sub‐inhibitory concentrations of thyme essential oil significantly reduced the growth rate compared to the control. Expression of all tested virulence genes was also reduced by the essential oil in a significant dose‐ and time‐dependent manner. As an example, decreased down‐regulation of *hil*A, *spv, sef*A and *inv*A as 1.7‐, 4.14‐, 2.92‐ and 1.04‐fold in 25% MIC and 6.42‐, 7.81‐, 4.4‐ and 3.75‐fold in 75% MIC was observed, respectively, after 24 h of incubation. Likewise, levels of transcription for *hil*A, *spv, sef*A and *inv*A were reduced 4.75‐, 6.95‐, 3.75‐ and 2.98‐fold after 18 h and 9.54‐, 8.81‐, 5.65‐ and 4.77‐fold, respectively, after 72 h in 75% MIC compared to the control.

**Conclusions:**

According to our data, aside from the growth inhibitory effect of thyme essential oil, the results of current study highlight the potential of thyme for reducing the transcriptional level of virulence genes and therefore the pathogenicity of *S*. *enteritidis*.

## Graphical abstract

The effects of thyme essential oil at sub‐inhibitory concentrations on bacterial growth and expression of some virulence genes in *Salmonella enteritidis* were assessed. The results showed that thyme essential oil significantly reduced the growth rate compared to the control. In addition, expression of all tested virulence genes was reduced by the essential oil in a significant dose‐ and time‐dependent manner.

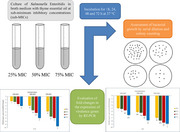

## 1 Introduction

Poultry products are among the principal animal‐source foods which are widely consumed at global level due to increasing consumer preferences for a high‐nutritional and healthy source of protein. Contaminated poultry meat and eggs are important vehicles for transmitting *Salmonella enterica* serotype Enteritidis (*S. enteritidis*) to humans. In poultry, infection with *S*. *enteritidis* is associated with clinical disease and mortality only in young birds during the first two weeks of age. Still, it yields asymptomatic intestinal pathogen carriage in older birds (Gast and Porter 2020). Intestinal colonization of *S*. *enteritidis* which primarily occurs in chicken ceca may result in carcass contamination at slaughterhouse (Stern et al. 2008). In layers, *S*. *enteritidis* invasion of intestinal cells leads to the systemic spread of the pathogen and contaminates the egg before oviposition. Therefore, controlling *S*. *enteritidis* infections in poultry flocks is an important measure for human health and the poultry industry (Tan et al. 2022).

It has been shown that *S*. *enteritidis* became highly resistant so that the multi‐drug resistant *Salmonella* serotypes have been developed as a result of non‐prudent use of antibiotics in poultry (Ghazaey and Mirmomeni 2012). Today, the investigation on natural antimicrobial compounds has gained more attention due to the public health concerns regarding the emergence of antimicrobial resistance in bacteria and potential risks of chemical residues in animal‐derived foods (Kollanoor‐Johny et al. 2012). In this regard, a number of treatments, including organic acids, oligosaccharides, bacteriophages, competitive exclusion bacteria, antibiotics, plant‐derived compounds and vaccines, have been used in poultry for reducing infections due to *Salmonella* spp. (Higgins et al. 2007; Borie et al. 2009; Penha Filho et al. 2010; Kollanoor‐Johny et al. 2012; Santos et al. 2013; El Baaboua et al. 2018).

Essential oils are produced as secondary metabolites by aromatic plants and can be extracted from different parts of plant structures such as leaves, bark, flowers, fruits, seeds and roots. These metabolites are often associated with plant defence against pathogenic agents and hence may possess antimicrobial properties (O'Bryan et al. 2015). Essential oils contain a wide variety of compounds; among them, phenols, terpenes, aldehydes and ketones are identified as effective antibacterial components (Nazzaro et al. 2013). The antibacterial activities of essential oils are usually exerted by multiple modes of action, with broad‐range and synergistic effects stemming from various components. In general, their action mechanism is based on denaturation and inactivation of membrane proteins and enzymes, destabilization of the electron flow and inhibition of the synthesis of biomolecules such as DNA, RNA, proteins and polysaccharides, targeting cell wall, cell membrane, genetic material, respiration and energy metabolism in bacterial cell (Herman, Bochenek, and Herman 2013). Field studies in poultry have reported that the addition of essential oils to poultry feed increases the feed conversion ratio and improves the growth performance of poultry by improving intestinal health and absorption of nutrients (Tiihonen et al. 2010; Giannenas et al. 2014).

Thyme (*Thymus vulgaris*) is indigenous to the Mediterranean region. It is grown commercially in Iran and many other countries for its leaves and production of essential oils and extracts as flavouring agent and herbal medicine (Abedini, Sahebkar, and Hassanzadeh‐Khayyat 2014). Thyme has been used in traditional medicine and is known to have a wide spectrum of biological properties, including anti‐inflammatory, antiviral, antibacterial and antiseptic agents (Kowalczyk et al. 2020). The antimicrobial activities of thyme essential oil against a diverse range of microorganisms have been reported in various studies (Abdollahzadeh, Rezaei, and Hosseini 2014; Borugă et al. 2014; Anžlovar, Likar, and Dolenc Koce 2017).

Previous studies have shown that *S*. *enteritidis* is the most prevalent serotype in poultry flocks of Iran and one of the most commonly isolated serotype from poultry in the world (Mayahi et al. 2017; Afshari et al. 2018; Shaji, Selvaraj, and Shanmugasundaram 2023). Many virulence genes are responsible for the pathogenicity of *S*. *enteritidis*. Among the main virulence genes of this pathogen, *hil*A is essential for the expression of the type III secretion system which injects bacterial effector proteins into the host cells. The *spv* encodes proteins involved in rapid growth and survival of *Salmonella* spp. within the host cells. *inv*A is necessary for epithelial invasion, whereas the *Salmonella*‐encoded fimbria (*sef*A) has a role in interaction between the bacterium and the macrophages (Borges et al. 2013).

Reducing the bacterial virulence mechanisms could prevent clinical disease of young chickens and the contamination of chicken carcasses or eggs due to diminished cecal colonization with *S*. *enteritidis* in chickens (Kollanoor‐Johny et al. 2012). The current study aimed to investigate the in vitro efficacy of thyme essential oil at sub‐inhibitory concentrations on the expression level of *inv*A, *hil*A, *sef*A and *spv* virulence genes critical for colonization and invasion of *S*. *enteritidis* in chickens. Moreover, the effect of thyme essential oil on the growth of *S*. *enteritidis* has also been evaluated.

## 2 Materials and Methods

### 2.1 Bacterial Strain and Essential Oil

*S*. *enteritidis* phage type 4 NIDO 76Sa88 Nalr strain was kindly provided as a lyophilized culture from the Faculty of Veterinary Medicine, Ghent University, Belgium. Steam distillated thyme essential oil was obtained from the Institute of Medicinal Plants (Alborz, Iran). Chemical compounds of the essential oil were identified by gas chromatography/mass spectrometry (GC/MS) (Agilent 6890‐5973, USA) equipped with helium gas as the carrier and a BPX5 capillary column (30 m × 0.25 mm ID × 0.25 µm film thickness). The components were detected by comparison of the retention time and mass spectra fragmentation with National Institute of Standards and Technology data (Sparkman 2005).

### 2.2 Minimal Inhibitory Concentration (MIC) Assay

MIC was determined using the broth microdilution assay. Twofold serial dilutions of essential oil were prepared between 1% and 0.015% (v/v) in tryptic soy broth (TSB) containing 5% (v/v) dimethylsulfoxide (DMSO) in a 96‐well polypropylene microtiter plate (Azizkhani et al. 2013). DMSO was used to increase the solubility of oil in the medium. The bacterial suspensions, with a turbidity of 0.5 McFarland, were made from overnight culture in Luria–Bertani (LB) broth and then added to each well at equal volume. The final volume in each well was 200 µL. Wells containing culture medium plus DMSO and bacteria with no essential oil added were considered control. The microplate was incubated at 37°C for 24 h in a shaker incubator. Bacterial growth was assessed by measuring 490 nm absorbance at the spectrophotometer (Stat Fax 2100, Awareness Technology, United States). The MIC value was defined as the lowest essential oil concentration that generated zero absorbance after 24 h of incubation.

### 2.3 Assessment of Bacterial Growth

TSB culture media were prepared with the addition of thyme essential oil at sub‐inhibitory levels (25%, 50% and 75% MIC) and 5% (v/v) DMSO. The culture media were then inoculated with *S*. *enteritidis* at 1.5 × 10^5^ (log = 5.17) CFU/mL concentration and were incubated at 37°C. The control culture contained 5% DMSO and inoculated bacteria with zero amount of essential oil. Serial dilutions were prepared from the TSB cultures incubated for 18, 24, 48 and 72 h, and counts of *S*. *enteritidis* were determined by spreading 0.1 mL of serial dilutions on TSA plates. The plates were incubated at 37°C for 24 h, then colonies were enumerated, and the results were expressed as log_10_ CFU/mL.

### 2.4 DNA Extraction and Amplification

*S*. *enteritidis* strain was subjected to DNA extraction by using the DNA extraction kit (Sinaclon, Iran) according to the manufacturer's guidelines. The extracted DNA was stored at −20°C until use. The presence of *inv*A, *hil*A, *sef*A and *spv* genes was evaluated by individual polymerase chain reactions (PCR) by using specific primer pairs (Table 1). PCR was performed in 25 µL volumes in a mixture containing 7.5 µL of distilled water, 1 µL of each primer, 3 µL of template DNA and 12.5 µL of a 2× PCR master mix (Sinaclon, Iran). The reactions were carried out on a thermal cycler (Bio‐Rad, United States) under the following conditions: initial denaturation at 94°C for 5 min, 35 cycles of denaturation at 94°C for 60 s, annealing at 60°C for 60 s and extension at 72°C for 1.5 min followed by a final extension step at 72°C for 7 min. The amplification products were analysed by agar gel electrophoresis on 1.5% agarose gels stained with safe stain to visualize bands in the gel document system (Bio‐Rad, United States).

**TABLE 1:** Primers sequences and amplified products for the targeted genes for *Salmonella enteritidis*.

Target gene		Primer sequence 5′‐3′	Amplified product (bp)	Reference
*inv*A	F	GTGAAATTATCGCCACGTTCGGGCAA	284	Borges et al. (2013)
	R	TCATCGCACCGTCAAAGGAACC		
*hil*A	F	ACGGCTCCCTGCTACGCTCA	401	Pan and Liu (2002)
	R	GCTCAGGCCAAAGGGCGCAT		
*sef*A	F	GCAGCGGTTACTATTGCAGC	310	Mirzaie et al. (2010)
	R	TGTGACAGGGACATTTAGCG		
*spv*	F	GCCGTACACGAGCTTATAGA	250	Mirzaie et al. (2010)
	R	ACCTACAGGGGCACAATAAC		

### 2.5 RNA Isolation and RT‐PCR

Total RNA extraction from *S*. *enteritidis* was performed after 18, 24, 48 and 72 h of incubation with sub‐inhibitory concentrations of thyme essential oil using the SinoPure Kit (Sinaclon, Iran) according to the manufacturer's instructions. The yield and quality of the extracted RNA were determined spectrophotometrically using 260 and 280 nm measurements (Boeco, Germany). The extracted RNA was subsequently reverse transcribed using a cDNA Synthesis Kit (SinaClon, Iran) according to the manufacturer's instructions. The obtained cDNA was stored at −70°C until use.

RT‐PCR reaction was prepared in a final volume of 20 µL, containing 10 µL of 2× Master Mix Green (Ampliqon, Denmark), 1 µL of each primer, 3 µL of cDNA and 5 µL of nuclease‐free water. 16S rRNA gene was selected as the reference gene for normalization of gene expression data. The primer pair used for 16S rRNA gene amplification was designed based on published GenBank *Salmonella* sequences using the Primer3Plus programme as follows: forward primer 5′‐CGGACGGGTGAGTAATGTCT‐3′ and reverse primer 5′‐GTTAGCCGGTGCTTCTTCTG‐3′. RT‐PCR was performed in the thermal cycler Rotor‐Gene 6000 (Corbett, Australia). Each reaction involved a pre‐incubation at 95°C for 4 min, followed by 40 cycles of 95°C for 15 s, 60°C for 30 s and elongation at 72°C for 30 s. RT‐PCR experiment included duplicate test samples. Cycle of threshold (CT) values, the cycle at which fluorescence dye exceeds a significant threshold, were obtained using Rotor‐Gene 6000 software version 1.7.27. CT information was used to calculate relative target and reference gene expression in treatment and control samples. Relative quantification of gene expression levels was calculated using the 2^−ΔΔ^ *^CT^* method (Livak and Schmittgen 2001).

### 2.6 Statistical Analysis

The data were statistically analysed by one‐way ANOVA approach with Scheffe post hoc test using SPSS Software v. 26 (IBM Analytics, New York, USA). Differences were considered significant at *p* < 0.05.

## 3 Results

### 3.1 Chemical Composition of Thyme Essential Oil

GC/MS analysis resulted in the identification of a mixture of terpenes composed predominantly of monoterpenes (95.48%), whereas sesquiterpenes represent less than 2% of the essential oils. Results indicated the presence of 29 various compounds in monoterpenes, with the oxygenated monoterpene thymol (37.61%) as the major compound. Other compounds present in significant amounts were monoterpene hydrocarbons p‐cymene (27.47%) and γ‐terpinene (10.04%). Some other oxygenated monoterpenes, including carvacrol (3.4%), linalool (2.46%) and borneol (2.03%), were also found (Table 2).

**TABLE 2:** Chemical composition of thyme essential oil identified by gas chromatography/mass spectrometry (GC/MS).

Component	KI^a^	Yield (%)	Component	KI	Yield (%)
**Monoterpenes**					
Tricyclene	926	0.07	Terpinen‐4‐ol	1177	0.8
α‐Thujene	930	1.48	p‐Cymen‐8‐ol	1182	0.11
α‐Pinene	939	1.18	α‐Terpineol	1188	0.27
Camphene	954	1.05	Thymol, methyl ether	1235	0.47
Sabinene	975	0.04	Carvacrol, methyl ether	1244	0.35
β‐Pinene	979	0.3	Geraniol	1252	0.1
Myrcene	990	1.4	l‐α‐Bornyl acetate	1285	0.33
α‐Phellandrene	1002	0.23	Thymol	1290	37.61
α‐Terpinene	1017	1.45	Carvacrol	1299	3.4
p‐Cymene	1024	27.47	Thymol acetate	1352	0.08
Limonene	1029	0.5	**Total**		95.48
1,8‐Cineole	1031	0.93	**Sesquiterpenes**		
γ‐Terpinene	1059	10.04	*E‐*Caryophyllene	1419	0.97
*cis*‐Sabinene hydrate	1070	0.57	γ‐Muurolene	1479	0.05
Terpinolene	1088	0.08	Germacrene D	1481	0.06
*p*‐Cymene	1091	0.05	γ‐Cadinene	1513	0.06
Linalool	1096	2.46	δ‐Cadinene	1523	0.11
*cis*‐p‐Menth‐2‐en‐l‐ol	1122	0.07	Spathulenol	1578	0.18
Camphor	1146	0.56	Caryophyllene oxide	1583	0.5
Borneol	1169	2.03	**Total**		1.93

^a^Kováts retention index.

### 3.2 Antimicrobial Activity Against *S*. *enteritidis*

The MIC value of thyme essential oil against *S*. *enteritidis* was 1.25 mg/mL. Bacterial growth rates at sub‐inhibitory levels of the essential oil as well as the control culture are given in Table 3. As shown in the table, the growth rate status of *S*. *enteritidis* was ascending over time in all culture groups. However, results indicated that incubation of *S*. *enteritidis* with sub‐inhibitory levels of thyme essential oil after 18–72 h of incubation significantly decreased the bacterial density by 2–4 log CFU/mL compared to the control (*p *< 0.05). Overall, 75% MIC was the most inhibitory treatment and reduced the cell density of the microorganism 1, 1.06 and 4.11 log CFU/mL at 24 h of incubation compared to 50% MIC, 25% MIC and the control, respectively.

**TABLE 3:** Colony counting (log_10_ CFU/mL) cultured at sub‐inhibitory levels of thyme essential oil against *Salmonella enteritidis*.

Experimental groups	Time (hours)			
	18	24	48	72
25% MIC	4.86 ± 0.03^f^	5.93 ± 0.05^e^	6.98 ± 0.04^d^	8.01 ± 0.08^c^
50% MIC	4.84 ± 0.03^f^	5.87 ± 0.02^e^	6.94 ± 0.04^d^	7.98 ± 0.02^c^
75% MIC	4.83 ± 0.03^f^	4.87 ± 0.008^f^	6.94 ± 0.03^d^	7.96 ± 0.03^c^
Control	6.86 ± 0.03^d^	8.98 ± 0.02^b^	11.03 ± 0.12^a^	11.07 ± 0.11^a^

*Note*: Values are means ± standard error of mean (SEM). Data not sharing a common letter (a–f) differ significantly at *p *< 0.05.

### 3.3 Effect on Gene Expression

PCR results revealed that *S*. *enteritidis* carried *inv*A, *sef*A, *hil*A and *spv* genes (data not shown). All of the investigated *S*. *enteritidis* virulence genes presented down‐regulation, and the mRNA expressions diminished progressively with an increase in exposure time and concentration of essential oil. However, the level of decrease in expression was higher in *hil*A and *spv* compared to *inv*A and *sef*A genes (Figure 1). As shown in the figure, significant differences were found in mRNA expressions between sub‐inhibitory concentrations and time points (*p *< 0.05). For example, decreased down‐regulation of *hil*A, *spv, sef*A and *inv*A as 1.7‐, 4.14‐, 2.92‐ and 1.04‐fold in the presence of 25% MIC and 6.42‐, 7.81‐, 4.4‐ and 3.75‐fold in the presence of 75% MIC were observed, respectively, after 24 h of incubation. As an example of the difference in gene expression at various time points, transcriptional levels of *hil*A, *spv, sef*A and *inv*A genes decreased 4.75‐, 6.95‐, 3.75‐ and 2.98‐fold after 18 h and 9.54‐, 8.81‐, 5.65‐ and 4.77‐fold after 72 h, respectively, in 75% MIC treatment compared to the control.

FIGURE 1Relative gene expression of *hil*A (A), *spv* (B), *inv*A (C) and *sef*A (D) in *Salmonella enteritidis* cultured with sub‐inhibitory concentrations of thyme essential oil for 18–72 h. Fold reduction of gene expression was reported in comparison to essential oil free culture as control. Different letters (a,b) show a statistical difference (*p *< 0.05).

**Figure:**
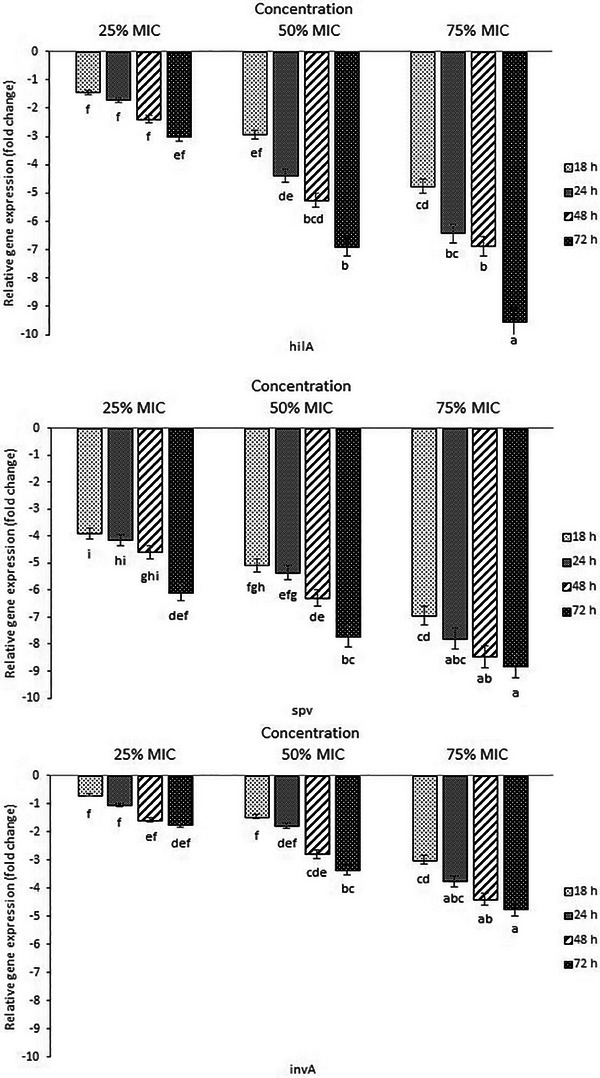

**Figure:**
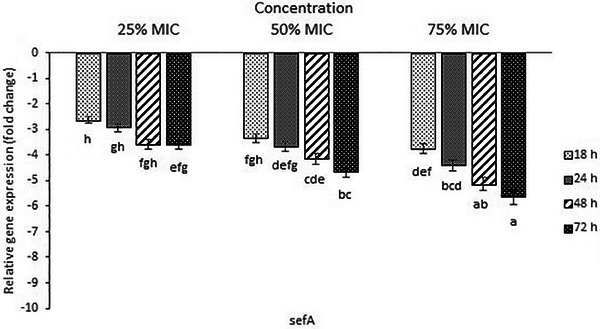

## 4 Discussion

*S*. *enteritidis* is the leading cause of food‐borne human diseases and economic losses in the poultry industry worldwide (Shah et al. 2017). It has been known that attachment, colonization and invasion of *S*. *enteritidis* in the chicken intestinal tract are essential for the emergence of systemic infection. In this regard, a potential strategy for controlling *S*. *enteritidis* is to decrease virulence factors associated with adhesion and invasion of the pathogen to host cells (Upadhyaya et al. 2013). Previous studies have indicated that plant‐derived essential oils can inhibit both the bacterial growth and the production of toxic bacterial metabolites (Nazzaro et al. 2013). As the nature of these compounds is multi‐bioactive component, the antimicrobial activity of essential oil is not associated with a unique mechanism of action. Hence, the risk for the emergence of resistant bacteria may be lower than many commonly used antibiotics with only a single target site (El‐Azzouny et al. 2018). The correlation between the chemical compositions of plant essential oils and their antimicrobial properties has been reported (Chouhan, Sharma, and Guleria 2017). Regarding thyme essential oil, the presence of oxygenated monoterpene (thymol) and monoterpene hydrocarbons (p‐cymene and γ‐terpinene) in the composition of the essential oil has been attributed to the antimicrobial activity (Borugă et al. 2014; Morshdy et al. 2022).

In general, essential oils are characterized by two or three major components which are present at higher amounts than their other trace constituents (Chouhan, Sharma, and Guleria 2017). Consistent with previous reports, results of the current study showed that the most abundant and principal components of thyme essential oil were thymol, p‐cymene and γ‐terpinene. In different studies, various amounts of 23%–60%, 8%–44% and 18%–50% for thymol, p‐cymene and γ‐terpinene, respectively, were reported (Borugă et al. 2014; Satyal et al. 2016; Aljabeili, Barakat, and Abdel‐Rahman 2018). The differences between our findings and other works are most likely attributed to the variation in plant growth environment and harvesting conditions along with the extraction processes. Thymol and carvacrol ingredients of thyme essential oil in the current study at 37.61% and 3.4% met the standard of minimum contents of 40% or 37%–55% thymol and 0.5%–5.5% carvacrol concentrations defined in European Pharmacopoeia X (Council of Europe 2019). It has been reported that thymol can negatively affect the proteins involved in energy metabolism, including ATP synthesis enzyme, and altered permeability of Gram‐negative bacteria such as *Salmonella* spp. cell membrane leading to the destruction and death of the bacterial cell (Di Pasqua et al. 2010; Nazzaro et al. 2013; Abdollahzadeh, Rezaei, and Hosseini 2014). Moreover, p‐cymene and γ‐terpinene exhibit their antibacterial effects by changing the plasma membrane lipid, which disrupts cytoplasmic membrane permeability and fluidity (Guimarães et al. 2019; Morshdy et al. 2022). However, as stronger antimicrobial activity was found for essential oils in comparison to that of their main components alone or their mixtures, synergistic effects of the minor constituents might also be important for exhibiting the biological activity of essential oils (Borugă et al. 2014).

Several studies assessed the MIC values of essential oils, including thyme oil on *Salmonella* spp. (Bajpai et al. 2012; Morshdy et al. 2022). Boskovic et al. (2016) reported that the sensitivity of *Salmonella senftenberg* to thyme essential oil and the MIC concentration of 640 µg/mL were found. Hoffman‐Pennesi and Wu (2010) reported the MIC value for thyme oil as 1.83 mg/mL, against *Salmonella typhimurium*. Based on the results of the current study, thyme essential oil had a 1.25 mg/mL MIC which was comparable to the literature values.

A number of studies have reported the inhibitory effects of plant‐derived compounds on the expression of bacterial virulence genes. For example, Hadjilouka et al. (2017) showed that expression of the key virulence genes of *Listeria monocytogenes* was down‐regulated by the exposure to lemongrass essential oil, and the increased amount of essential oil was more effective in the down‐regulation of gene expression. Azizkhani et al. (2013) reported that exposure of *Staphylococcus aureus* to sub‐inhibitory levels of *Zataria multiflora* essential oil significantly decreased the expression of staphylococcal enterotoxin‐related genes. Moreover, in *Escherichia coli*, bacterial genes related to toxin secretion, motility and adhesion to enterocytes were down‐regulated by thymol, carvacrol and eugenol (Bonetti et al. 2020). In agreement with previous studies, our data revealed that thyme essential oil at sub‐inhibitory concentrations could lower the expression level of *S*. *enteritidis* virulence genes in a dose‐ and time‐dependent manner compared to non‐treated control. These suppressing effects were more remarkable for *hil*A and *spv* than *inv*A and *sef*A genes. Down‐regulation in the expression of these virulence genes is associated with reduced epithelial invasion and bacterial growth and survival of *Salmonella* spp. within the host cells (Borges et al. 2013). Similar observations were reported by Giovagnoni et al. (2020) and Morshdy et al. (2022) as a significant down‐regulation of the main virulence genes of *S. typhimurium*, including *hil*A and *inv*A upon the treatment with thymol, carvacrol and essential oils from garlic and thyme. El‐Azzouny et al. (2018) reported that essential oils from garlic (*Allium sativum*) and Zataria (*Z. multiflora*) were effective in the down‐regulation of *Salmonella* membrane proteins encoding genes, *sop*B and *mgt*C which are associated with the virulence. In another study, down‐regulation in the expression of genes involved in *S*. *enteritidis* colonization in chicken oviduct and survival in macrophages by carvacrol, thymol and eugenol was reported (Upadhyaya et al. 2013). There is clear evidence that the presence of thymol in the environment of *Salmonella* carries the effects of an oxidative stress on the bacterial cell and the microorganism reacts to the induced stress (Di Pasqua et al. 2010). Hence, modifications of gene expression towards increased transcription level of more survival‐associated genes might be required to maintain or improve the ability of bacteria to grow in the presence of toxic substances.

Our experiment revealed that virulence gene expression level in bacterial cell exposed to the essential oil was reduced despite the increase of *S*. *enteritidis* counts over time regardless of the presence or amounts of the essential oils in the culture medium. Consequently, it could be hypothesized by the results of our study that the observed reduction in virulence gene expressions was attributed to the direct effect of essential oil and was not secondarily to the growth inhibition. Similar findings were also made by Azizkhani et al. (2013), who showed progressive inhibitory effects of *Z. multiflora* essential oil on the expression of enterotoxin‐codded genes of *S. aureus* with concurrent increasing of bacterial growth rate by extending incubation time.

## 5 Conclusion

Based on our findings, thyme essential oil at sub‐inhibitory concentrations exhibited significant antibacterial activity against *S*. *enteritidis*. As essential oils have many kinds of chemical components, there is more than one mechanism behind their antibacterial activity. One of the mechanisms of action against *S*. *enteritidis* may be the destruction of the cell wall and membrane, leading to increased membrane permeability and loss of vital intracellular contents like proteins, sugars and genetic materials. In addition, as a support for another underlying mechanism for the antimicrobial action of essential oils, our data revealed that down‐regulation of tested virulence genes, which are involved in the cecal colonization, intracellular survival and invasion of *S*. *enteritidis* in chicken, occurred by the effect of thyme essential oil. Results of the current study suggested that down‐regulation of virulence genes was time and dose‐dependent. Significant inhibition of growth and transcription of virulence genes of *S*. *enteritidis* by thyme essential oil compared to non‐treated control may highlight its potential for reducing the pathogenicity of *S*. *enteritidis* and controlling salmonellosis in broiler chicken.

## Author Contributions

Conceptualization: Sara Mirzaie and Mohammad Hassanzadeh. Investigation: Faezeh Rahimi Pirmahalle. Methodology: Sara Mirzaie, Mohammad Hassanzadeh and Ramak Yahyaraeyat. Writing–original draft preparation: Sara Mirzaie. Writing–review and editing: Jamshid Razmyar. Formal analysis: Sara Mirzaie.

## Ethical Statement

The authors confirm that the ethical policies of the journal, as noted on the journal's author guidelines page, have been adhered to. No ethical approval was required as no animal was used for the production of data in current study.

## Conflicts of Interest

The authors declare no conflicts of interest.

### Peer Review

The peer review history for this article is available at https://www.webofscience.com/api/gateway/wos/peer-review/10.1002/vms3.70088.

## Data Availability

The data that support the findings of this study are available from the corresponding author upon reasonable request.
